# Do Diet and Lifestyles Play a Role in the Pathogenesis of NMSCs?

**DOI:** 10.3390/nu12113459

**Published:** 2020-11-11

**Authors:** Nevena Skroza, Ilaria Proietti, Anna Marchesiello, Salvatore Volpe, Veronica Balduzzi, Nicoletta Bernardini, Patrizia Maddalena, Alessandra Mambrin, Simone Michelini, Ersilia Tolino, Giuseppe La Torre, Concetta Potenza

**Affiliations:** 1 Dermatology Unit “Daniele Innocenzi”, Department of Medical-Surgical Sciences and Bio-Technologies, Sapienza University of Rome, Fiorini Hospital, Polo Pontino, 04019 Terracina, Italy; nevena.skroza@uniroma1.it (N.S.); anna.marchesiello90@gmail.com (A.M.); sal.volpe@hotmail.it (S.V.); veronica.balduzzi@gmail.com (V.B.); nicoletta.bernardini@libero.it (N.B.); patrizia.maddalena@gmail.com (P.M.); mambrinalessandra@gmail.com (A.M.); simo.mik@hotmail.it (S.M.); ersiliatolino@gmail.com (E.T.); concetta.potenza@uniroma1.it (C.P.); 2 Department of Public Health and Infectious Diseases, Sapienza University of Rome, 00161 Rome, Italy; giuseppe.latorre@uniroma1.it

**Keywords:** NMSC, lifestyle, smoking, alcohol, diet

## Abstract

Background and Aims: Literature highlights the role of risk factors like age, body mass index (BMI), tobacco smoking, alcohol intake and diet in the pathogenesis of several cancer types but little is known for non-melanoma skin cancers (NMSC). The aim of this epidemiological study was to evaluate the correlation between modifiable risk factors (BMI, metabolic panel, diet, lifestyle, medical history) and not modifiable risk factors (gender, age) and NMSC development. Methods: From February 2018 to September 2019, 162 patients affected by NMSC were compared to a group of 167 controls. A univariate and multivariate analysis was conducted to elaborate the data collected through face-to-face interviews. Results: While our evidence did not always reach statistical significance, NMSC study group patients exhibited high rates of analyzed risk factors (male gender aging over 55 years, high BMI, reduced physical activity) compared to the control group. Conclusions: Our study indicates that practicing more than 30 min of physical activity daily could be a protective factor against the NMSC onset. Other risk factors were not correlated with NMSC, but more evidence is needed to establish a possible link.

## 1. Introduction

The incidence of non-melanoma skin cancer (NMSC), including predominantly basal cell carcinoma (BCC) and squamous cell carcinoma (SCC), has risen significantly among white populations, sparking an increasing scientific interest in these types of tumors [1,2].

Genetic factors and behavioral changes are known to promote skin cancer.

However, high frequency of UV radiation (UVR) exposure remains the predominant causative agent, altering cell genetic and immunological profile, leading to DNA damage, oxidative stress, free radical production and photo aging [3].

An additional well-established risk factor for NMSC includes Fitzpatrick I-II skin type. 

Modifiable lifestyle risk factors, such as diet, exposure to exogenous hormones, alcohol intake, and tobacco smoking, generally noted to be linked to carcinogenesis, have not definitively been confirmed as risk factors for NMSC.

Recently, extensive research has highlighted the role of correct eating habits in controlling skin carcinogenesis and tumor growth in other models; nevertheless, a comprehensive immunological evaluation implicated in this relationship is still pending [4].

Moreover, obesity has increased alarmingly in the Western world during recent decades, becoming a risk factor for several cancers [5].

However, evidence for an association between obesity and malignant melanoma (MM) and non-melanoma skin cancer (NMSC) has not been fully established yet.

Currently, results from a large meta-analysis showed that males with high body mass index (BMI) had a high risk for MM, whereas this association was not found among females [6].

Fewer studies have addressed the associations between BMI and risk for NMSC but the results are conflicting [7].

In order to investigate how much lifestyle affects the risk of developing NMSC, we conducted an epidemiological study evaluating the metabolic syndrome indices, eating behavior, smoking habits, alcohol consumption and physical activity in NMSC patients in correlation with a control study group.

## 2. Materials & Methods

From February 2018 to September 2019, a community-based case-control was carried out enrolling 162 cases of NMSC outpatients of a dermatological ambulatory service and 167 clinically healthy NMSC-free subjects, as controls.

Data collection was carried out through structured face-to-face interviews. 

Primarily, anamnestic data were collected regarding age, gender, work activity, health status and medications.

The second part of the interview concerned the dietary habits.

Normal eating habits of participants were assessed using the validated food frequency questionnaire (FFQ), which included 12 items corresponding to the 12 elemental characteristics of the Mediterranean diet: carbohydrates, vegetables, fruit, milk, extra virgin olive oil, white meat, red meat, sausages, fish, eggs, legumes and sweets [8]. 

For each participant, a score was constructed by adding the scores obtained for the 12 groups of foods. Adherence to the Mediterranean diet was assessed by a score created by Trichopoulou et al. [9].

Finally, participants were asked about their smoking habits and daily physical activity (for more or less than 30 min).

Furthermore, BMI was calculated for each patient complete with a metabolic panel (glycemia, cholesterolemia and triglyceridemia). 

## 3. Statistical Analysis

The statistical analysis was carried out using SPSS (IBM Corp. Released 2017. IBM SPSS Statistics for Windows, Version 25.0. Armonk, NY, USA: IBM Corp), release 25.0.

The univariate analysis was conducted using the chi-square test in order to compare the cases and controls for different dichotomous variables. 

The multivariate analysis was performed using a logistic model. The results are presented as odd ratio (OR) (95% CI). Two types of model were created, the first model including all the variables (full model), and the second one using a stepwise approach (backward elimination).

The significance level was set at *p* value < 0.05.

Since the variables used for the multivariate analysis were not continuous, no multicollinearity was present.

No missing data were present.

Composite scores based on the items were constructed.

## 4. Results

The survey included 326 patients: 162 (96 males and 66 females) affected by NMSC (BCC, actinic keratosis (AK), SCC), with a mean age of 68 years (range 36–95); the control group consisted of 164 subjects not suffering from skin cancers (82 males and 82 females), with a mean age of 57 years (range 32–88) (Table 1 and Table 2). Male gender and age < 55 years were found to be associated with a higher risk of developing NMSC (*p* value > 0.05). BMI was used to categorize each person as underweight, normal weight, overweight or obese: the NMSC group was 1% underweight, 31% normal weight, 44% overweight, 20% first grade obesity, 4% second grade obesity and 0% third grade obesity; the control group was 1% underweight, 52% normal weight, 29% overweight, 15% first grade obesity, 2% second grade obesity and 1% third grade obesity. The condition of obesity or overweight was significantly higher in the NMSC group (*p* value < 0.001). There were no statistically significant differences between the two groups in the frequency and the amount of consumption of the aliments examined. Nevertheless, cold meat (*p* value = 0.055) and sugary drinks (*p* value = 0.076) were found to be widely consumed in the NMSC group. As far as concerns the Mediterranean diet score, levels of the score equal to or over 10 were associated with a lower risk of NMSC (*p* = 0.056).

Concerning the metabolic panel assessment (glycemia, cholesterolemia and triglyceridemia), blood tests were over the limits in 76 NMSC patients (46.9%) and in 74 (44.3%) control study group subjects. This difference was not statistically significant (*p* value = 0.636), although considerably high levels of glycemia were observed in the NMSC group. In the NMSC group 83 patients (51.2%) declared themselves to have never smoked and 79 patients (48.8%) declared themselves to be smokers or former smokers. In the control group, on the other hand, 96 patients (58.5%) reported never having smoked and 68 patients (41.5%) claimed to be smokers or former smokers. These data were not statistically significant (*p* value = 0.185). Differences in the frequency of alcohol consumption were minimal and not statistically significant. Among NMSC patients, only 33 (20.4%) claimed to practice physical activity for more than 30 min daily, as opposed to 60 patients (36.6%) from the control group.

## 5. Discussion

Skin cancer represents one of the most common worldwide malignancies and its incidence exhibits no signs of plateauing [10].

Whereas different types of cancers are causally related to a variety of endogenous and exogenous modifiable risk factors, the concept that lifestyle behavior may play a role in modulating cancer incidence has become largely investigated, recently, upon indirect epidemiologic evidences.

However, few studies have found correlations of dietary fat intake with NMSC incidence [11].

Commonly, dietary history and lifestyle habits questionnaires and surveys are widely used procedures for collecting epidemiological data about this relation. Nevertheless, with regard to NMSC, only a few epidemiological investigations found no association between its incidence and dietary intake [12,13].

Through the interviews performed, we observed a suggestive pattern of elevated risk of NMSC in male subjects, aged over 55 years. This trend reflects the recent incidence data [14].

No statistically significant correlation has been observed between the foods investigated, neither those typical of the Western diet nor those characteristic of the Mediterranean diet.

Despite the Mediterranean diet showing a favorable impact on some chronic inflammatory skin conditions such as acne, rosacea, hidradenitis suppurativa, psoriasis as well as certain malignancies, presumably due to its anti-inflammatory and antioxidant effects, more high-quality research is needed to verify a presumable correlation with NMSC [15,16,17].

Physical activity influences the risk of several types of malignancies. There is much evidence sustaining the role of regular physical activity in reducing risk of colorectal cancers, breast cancers, endometrial cancers, testicular cancers and minorly, lung and pancreas tumors [18,19,20].

Suggested biological pathways by which physical activity may influence cancer risk include changes in hormones, growth factors, inflammatory cytokines, and immune function.

Regarding skin cancer, physical activity explicates its beneficial effects by improving immune function, increasing detoxification and DNA repair, thus reducing sun exposure-induced DNA damage [21].

Nevertheless, some authors indicate a potential positive association between outdoor physical activity and keratinocyte cancer, due to high UV radiation exposure levels while performing outdoor sports [21].

Other studies also highlight existing sex differences in the association between recreational physical activity and the development of SCC due to more outdoor exercise, less clothing, and less use of sunscreens in men than women [22].

Our data suggest a possible protective effect of physical activity, for at least 30 min daily, on NMSC onset. In fact, among 162 NMSC patients, only 33 (20.4%) claimed to practice physical activity for more than 30 min daily, as opposed to 60 subjects (35.9%) of the control group.

The limit of this observation is attributable to the fact that these data were not adjusted for outdoor sun exposure or any other risk factor pertaining to sun exposure.

Although not statistically significant, higher BMI was observed in NMSC patients than in the control group. In addition, the obesity condition was significantly increased in the NMSC study population (*p* value <0.0001).

The BMI of adults and the global obesity prevalence is trending higher. Numerous epidemiologic studies reported, constantly, that severe and morbid obesity are associated with elevated risks of adverse health outcomes and highlighted a high BMI as a potential risk factor for many types of malignancies [23].

Recent evidence suggests that adipocytes could play an important role in the proliferation of cancers, although the mechanism underlying promotion of carcinogenesis is not fully established [24].

Specific to NMSC, the association with high BMI is controversial, since various studies reported an inverse relationship, while others have not found significant results [25].

Regarding skin cancer, the conflicting association with being overweight or obese could have both behavioral and biological explanations: on one side, presumably, overweight subjects are less likely to be exposed to UV light in public settings, the primary risk factor for skin cancer [26,27].

On the other side, a potential mechanism is due to the alterations in hormones and growth factors induced by high caloric intake, that lead either to height or to an increased number of cells that could potentially have an abnormal proliferation.

In support of this evidence, studies conducted in obese leptin-deficient mice skin tissue revealed stronger inflammatory response to UV radiation and greater oxidative stress resulting in altered cellular signaling [28].

Therefore, our observation of a suggestive positive association between high BMI and NMSC development could sustain this position.

## 6. Limitations of the Study

Regarding the role of physical activity as a presumable protective factor against NMSC development, the limit of our observation is attributable to the fact that our data were not adjusted for outdoor sun exposure or any other risk factor pertaining to sun exposure.

## 7. Conclusions

In these past years, NMSCs have acquired a clinical relevance that parallels their steadily increasing incidence.

Moreover, many patients often exhibit two or more types of NMSC simultaneously.

In fact, the main limitation of our study is the lack of stratification regarding the three main epithelial tumors (AK, BCC, SCC), as they often coexist in the same subject.

As in all malignancies, for NMSC it is advisable to investigate possible modifiable risk factors in order to act promptly on their prevention.

Our study has revealed the existence of some risk factors such as high BMI, male gender aging over 55 years and lack of physical activity, as potentially related to a high risk of developing NMSC.

Although the correlation of these factors and the development of NMSC is still controversial, additional studies are necessary to confirm or disprove our hypotheses.

## Figures and Tables

**Table 1:** Univariate analysis table.

	Cases	Controls	*p* Value
Age			<0.001
<55 years	16 (9.9)	0 (0)	
≥55 years	146 (90.1)	167 (100)	
Sex			0.03
Females	65 (40.1)	94 (56.3)	
Males	97 (59.9)	73 (43.7)	
Tobacco smoking			0.142
Non-smokers	83 (51.2)	99 (59.3)	
Smokers/ex-smokers	79 (48.8)	68 (40.7)	
Metabolic panel			0.636
Normal	86 (53.1)	93 (55.7)	
Not normal	76 (46.9)	74 (44.3)	
Body mass index			<0.001
Normal	52 (32.1)	89 (53.3)	
Overweight/obesity	110 (67.9)	78 (46.7)	
Physical activity			0.002
<30 min daily	129 (79.6)	107 (64.1)	
≥30 min daily	33 (20.4)	60 (35.9)	
Mediterranean diet score ≥ 10			0.056
<10 (reference)	157 (96.9)	151 (92.1)	
≥10	5 (3.1)	13 (7.9)	

**Table 2:** Multivariate analysis table.

	Full Model		Backward Elimination Model	
	OR	CI 95%	OR	CI 95%
Sex				
Females	0.59	0.36–0.96	0.59	0.37–0.94
Males (reference)	1		1	
Tobacco smoking				
Non-smokers (reference)	1			
Smokers/ex-smokers	1.01	0.62–1.63		
Metabolic panel				
Normal (reference)	1			
Not normal	0.80	0.50–1.28		
Body mass index				
Normal (reference)	1		1	
Overweight/obesity	1.94	1.20–3.15	1.90	1.18–3.05
Physical activity				
<30 min daily (reference)	1		1	
≥30 min daily	0.50	0.29–0.75	0.52	0.31–0.86
Mediterranean diet score > 10				
<10 (reference)	1	0.13–1.14	1	0.12–1.05
≥10	0.38		0.36	
